# Masquelet technique with radical debridement and alternative fixation in treatment of infected bone nonunion

**DOI:** 10.3389/fsurg.2022.1000340

**Published:** 2022-10-06

**Authors:** Xuanzhe Liu, Hong Sung Min, Yimin Chai, Xiaowei Yu, Gen Wen

**Affiliations:** Shanghai Sixth People's Hospital Affiliated to Shanghai Jiao Tong University School of Medicine, Shanghai, China

**Keywords:** infected bone nonunion, Masquelet technique, IL-6, radical debridement, alternative fixation

## Abstract

**Background:**

Infected bone nonunion is the toughest problem in fracture-related infection, leading to high disability and recurrence. The aim of this study was to evaluate the effectiveness of the Masquelet technique with radical debridement and alternative fixation in the management of infected bone nonunion.

**Patients and Methods:**

A retrospective study of prospectively collected data in two trauma centers was performed from 2016 to 2020. Patients diagnosed as infected bone nonunion were included in this study. The initial implant was removed and all patients received a two-stage Masquelet procedure with radical debridement and alternative fixation. The disappearance of inflammatory manifestations and regression of infection indicators (such as interleukin-6 (IL-6), C-reactive protein, white blood cell count) to the normal range were regarded as radical debridement. The alternative fixation depended on local soft tissue conditions. Results were evaluated according to clinical and radiographic assessment and patient satisfaction.

**Results:**

A total of 23 patients were included in our study. Six of them received internal fixation, while the other 17 received external fixation. Of the 23 cases, 21 were successfully reconstructed without infection recurrence, except 2 reinfected cases. Mean full weight bearing time was 6.6 months follow-up post last surgery. Out of the 23, 20 cases had satisfactory functional outcomes without additional bone or soft tissue comorbidities. Discrepancies in leg length and joint stiffness were observed in three cases and marked as unsatisfied results.

**Conclusions:**

Infected bone nonunion can be successfully managed using the Masquelet technique under radical debridement combined with an alternative fixation method.

## Introduction

Infected bone nonunion (IBN) is a fracture-related infection (FRI) with or without a fixation device, defined as a fracture without bone healing longer than 6 months (1). The incidence of FRI is as high as 30% in open fractures. Among all FRI, IBN is the toughest problem in FRI, leading to high amputation and recurrence rate (2) and carrying a serious socioeconomic burden (3). The pathogenesis of IBN has been attributed to multiple mechanisms of pathogen infection (4) and fracture-end instability (5). A vicious cycle between osseous instability with ongoing deteriorated soft tissue conditions and osteolysis renders a bacteria-friendly environment and hinders infection eradication. These mechanisms of IBN shed more light on whether the debridement surgical technique is performed thoroughly and the stability of a suitable fixation method.

The Masquelet technique, also known as the induced membrane technique, has been accepted as a two-stage procedure to treat bone defects. The first stage is by filling up bone defects with polymethyl methacrylate (PMMA) cement. PMMA cement is used as a spacer to eliminate dead ends, a local antibiotic delivery system, and a bioreactor chamber to stimulate osteogenesis. The second stage is the osteosynthesis procedure allowing the mixture of autologous bone grafting and allograft. The secret to the success of the Masquelet technique is the radical debridement, as is highlighted in the treatment of infected nonunion.

The gold standard of radical debridement remained still in debate due to the fact that sometimes infection existed lack of clinical signs and negative results of pathogen culture (3). Although advanced imaging technique, like PET-CT, has already been introduced to diagnose IBN, the inconvenience of the examination and the radioactive properties limits its use. Systemic immunoresponses evaluation of Cierny and Mader was instructive and inspiring in the diagnostic and treatment of infected bone nonunion (6). Regression of serological infection indicators [such as interleukin-6 (IL-6), C-reactive protein (CRP), white blood cell count (WBC)] to the normal range was regarded as one of the diagnostic tests with high sensitivity to radical debridement. IL-6 is a novel potential inflammatory marker gaining increasing interest, other than conventional WBC, CRP, and erythrocyte sedimentation rate (ESR). IL-6 in serum or synovial fluid has shown a high specificity in diagnosis of periprosthetic joint infection (7). In IBN treated by the Masquelet technique, IL-6 might also be used as a feasible indicator to decide whether the recurrent infection has been eliminated.

Another vital pathogenic factor influencing infected bone nonunion was the biomechanical status of fracture end osseous tissue. More and more studies have shown the removal of infected implantation might aggravate local infection (8). Therefore, adequate fixation no matter whether internal or external fixation should be suggested in IBN. Commonly, external fixators have been widely used in IBN. The Ilizarov technique combined with the Masquelet technique has been verified effective in the management of bone defects in the infected nonunion of the tibia (9). However, the internal fixation could also be considered, as long as the fracture ends were thoroughly debrided and benign soft tissue coverage. Hence, the alternative fixation of IBN is also an interesting issue to investigate.

In our study, we hypothesized that the Masquelet technique with radical debridement and alternative fixation can effectively treat infected bone nonunion.

## Patients and methods

This study was approved by the Institutional Ethical Review Board of Shanghai Jiaotong University Affiliated Sixth People's Hospital [IRB No.: 2016-88-(4)]. All of the patients enrolled in this study signed written informed consent. The study methods were in accordance with the Declaration of Helsinki.

### Inclusion criteria and exclusion criteria

Between January 2016 and December 2020, 23 patients diagnosed with infected bone nonunion were treated using the Masquelet technique combined with alternative fixations in our two trauma centers. Patients were aged from 18 to 75 years. The demographic information of patients was demonstrated in Table 1. Patients who underwent segmental bone defect (>1.5 times diameter of bone defect) or critical size soft tissue defect were not included in this study. Patients with pathological fractures or noninfected nonunion were excluded.

**Table 1:** Demographics and detailed information of the included cases.

Case number	Sex	Age	Etiology	Gustilo–Anderson classification of initial trauma	Times of previous surgery	Site of bone nonunion	Size of bone defect	Pathogen of bacteria culture	Type of fixation	Full weight bearing (months)	Complication	Results
1	M	47	CI	IIIB	2	Middle 1/3 tibia	3.7	*S. aureus*	Ex/Ring	7	NA	Satisfactory
2	M	49	RTA	II	2	Distal 1/3 tibia	4	MRSA	Int	6	NA	Satisfactory
3	M	55	RTA	I	2	Proximal 1/3 tibia	2.8	*Escherichia coli*	Ex/Ring	6	Joint stiffness	Unsatisfactory
4	F	37	RTA	IIIB	2	Middle 1/3 tibia	3.4	*Klebsiella pneumoniae*	Int	5	NA	Satisfactory
5	M	43	CI	IIIA	4	Middle 1/3 tibia	4.3	MRSA	Ex/Ring	8.5	NA	Satisfactory
6	M	56	RTA	II	4	Distal 1/3 tibia	3.6	*S. aureus*	Ex/Ring	8	NA	Satisfactory
7	M	74	RTA	II	3	Middle 1/3 tibia	4	MRSA	Ex/ML	7	NA	Satisfactory
8	M	47	RTA	IIIA	3	Distal 1/3 tibia	2.5	MRSA	Ex/Ring	5	NA	Satisfactory
9	M	34	RTA	II	4	Proximal 1/3 tibia	3.6	*Pseudomonas aeruginosa*	Int	6	NA	Satisfactory
10	M	61	RTA	IIIA	3	Distal 1/3 tibia	3.8	*S. aureus*	Ex/ML	6.5	NA	Satisfactory
11	M	51	RTA	IIIC	3	Distal 1/3 tibia	4.5	MRSA	Ex/Ring	12	Reinfected; LLD 2cm	Unsatisfactory
12	F	38	RTA	IIIB	2	Distal 1/3 tibia	3	*E. coli*	Ex/LCP	6	NA	Satisfactory
13	M	46	II	IIIB	2	Distal 1/3 femur	2.7	*P. aeruginosa*	Ex/ML	5	Reinfected; Joint stiffness	Unsatisfactory
14	M	42	RTA	II	4	Middle 1/3 humerus	3.5	MRSA	Ex/ML	7.5	NA	Satisfactory
15	M	49	RTA	I	3	Distal 1/3 tibia	3.3	*S. aureus*	Ex/Ring	7	NA	Satisfactory
16	M	45	RTA	IIIB	2	Middle 1/3 humerus	3.8	*P. aeruginosa*	Int	5.5	NA	Satisfactory
17	M	48	RTA	IIIA	2	Proximal 1/3 tibia	2	*S. aureus*	Ex/Ring	6	NA	Satisfactory
18	M	46	RTA	IIIB	3	Proximal 1/3 tibia	3.6	*E. coli*	Int	5	NA	Satisfactory
19	M	42	CI	IIIA	3	Middle 1/3 humerus	4.1	*P. aeruginosa*	Ex/ML	8	NA	Satisfactory
20	M	59	RTA	I	3	Middle 1/3 tibia	3.2	MRSA	Int	4.5	NA	Satisfactory
21	F	45	RTA	IIIB	2	Proximal 1/3 tibia	3.4	*E. coli*	Ex/Ring	6	NA	Satisfactory
22	M	49	RTA	IIIA	4	Distal 1/3 tibia	2.8	*S. aureus*	Ex/LCP	5	NA	Satisfactory
23	M	56	CI	IIIB	3	Proximal 1/3 tibia	4	MRSA	Ex/Ring	10	NA	Satisfactory

RTA, road traffic accident; CI, crush injury; II, iatrogenic infections; Ex, external fixation; Int, internal fixation; LCP, locking compression plate; ML, monolateral fixator; MRSA, methicillin-resistant Staphylococcus aureus; LLD, leg length discrepancy.

### Preoperative assessment

Preoperative data of all patients were obtained from His system in-ward. Comorbidities such as smoking and alcohol abuse history, diabetes, or cerebrovascular disease history were recorded in preoperative data. The bacterial culture was obtained by a swab culture during the first debridement surgery. The serum infection biomarkers were dynamically monitored after being in-ward, including WBC, CRP, ESR, and IL-6. Preoperative imaging evaluation was performed by experienced radiologists.

### Surgical procedure

#### First stage

The first stage procedure included removal of the initial fixation and radical debridement of the infection site. The vacuum sealing drainage (VSD) technique was applied to achieve effective drainage of the seriously infected wound and promote newborn granulation tissue growth of soft tissue if needed. The implantation of an antibiotic cement spacer was applied to fill up the dead space. A 2 g vancomycin was mixed into 40 g of cement to target the common spectrum bacteria by empirical application. The condition of soft tissue coverage was evaluated for further choices of fixation method. If the soft tissue coverage was good enough for further internal fixation implant, the cast was used for temporary fixation of the limb. Otherwise, the definite external fixation with ring was applied during the first stage to achieve bone stabilization.

#### Second stage

In our studies, the interval time between the two stages of surgery was 4–6 weeks as recommended by Masquelet techniques (10). The second stage procedure was performed until the biomarkers of infection (including IL-6, CRP, and ESR) were reduced to the normal range. The second stage included removal of residual cement with cautious protection of induced membrane, recanalization of bone ends, sufficient bone graft with iliac autologous bone grafts (ABGs), and allograft and osteosynthesis of the nonunion bone loss by taking advantage of the induced membrane. The cast would be replaced by internal fixation, or the external fixation would be adjusted to appropriately pressurize bone ends to stabilize bone ends.

### Follow-up assessment

All patients in both centers received standardized postoperative care, including sterile dressing change of wound, consecutive standardized application of systemic antibiotics, regular postoperative radiography, physical therapy rehabilitation, and external fixation care if needed. Patients were followed up at 3, 6, 12, 18, and 24 months after definite surgery. Patients who received external fixator would undergo a dynamic motorization period after bone healing to accelerate bone consolidation. The evaluation was conducted by an orthopedist to assess for major complications. Major complications were identified as: soft tissue coverage failure, recurrent infection after definite surgery of fixation, fixation failure of internal or external fixation, refracture without secondary injury, and residual deformities required orthopedic surgery.

## Results

The demographic features of 23 patients were listed in Table 1. All patients have a history of 2.8 ± 0.8 times of surgeries. Eighteen patients underwent traffic accidents, others including four crush injuries and one iatrogenic infection. The most common pathogen was *Staphylococcus aureus*, which was found in 14 cases. Eight of 14 were found to be methicillin-resistant *Staphylococcus aureus* (MRSA). The mean length of partial or total bone defect was 3.5 ± 0.6 cm.

### Clinical results

Statistical results were summarized in Table 2. Twenty-one of the 23 cases were successfully reconstructed without infection recurrence, except 2 reinfected cases (91.3%). Nineteen cases were located at the tibia, 3 cases at the humerus, and 1 case at the femur. Six of 23 cases received internal fixation, while the other 17 received external fixation with a ring fixator, monolateral fixator, or locking plate. Two of them strongly refused to use external fixation due to previous unhappy surgical experiences. External fixation of the locking plate was used instead. The two reinfected patients received external ring fixation and achieved remission after re-debridement. The mean time from definite surgery to full weight bearing was 6.6 months. Twenty out of 23 cases had satisfactory functional outcomes without additional bone or soft tissue comorbidities. Discrepancies in leg length and stiff joints were observed in three cases and marked as unsatisfied results. Three patients were treated conservatively.

**Table 2:** Summary of results.

	*N*	%
Total number of cases	23	100%
Age (years)	34–74 years	Average 48.6 years
Gender	Males: 20	86.9%
	Females: 3	13.0%
Previous surgeries	2.8 times	100%
Etiology	RTA: 18	78.2%
	CI: 4	17.3%
	II: 1	4.3%
Bacterial culture	MRSA: 8	34.7%
	*S. aureus*: 6	26.1%
	*E. coli*: 4	17.4%
	*P. aeruginosa*: 4	17.4%
	*K. pneumoniae*: 1	4.3%
Site of bone defect	Proximal 1/3 tibia: 6	26.1%
	Middle 1/3 tibia: 5	21.7%
	Distal 1/3 tibia: 8	34.8%
	Distal 1/3 femur: 1	4.3%
	Middle 1/3 humerus: 3	13.0%
Size of bone defect	2–4.5 cm	Average: 3.5 cm
Type of fixation	Internal: 6	26.0%
	External:17	73.9%
	Ring: 10	43.4%
	Monolateral: 5	21.7%
	LCP: 2	8.7%
Full weight bearing (months)	5–12 months	Average: 6.6
Results	Satisfactory: 20	86.9%
	Unsatisfactory: 3	13.0%
Complications	Recurrent infection: 2	8.6%
	2 cm LLD: 1	4.3%
	Joint stiffness: 2	8.6%

MRSA, methicillin-resistant Staphylococcus aureus; RTA, road traffic accident; CI, crush injury; II, iatrogenic infections; LCP, locking compression plate; LLD, leg length discrepancy.

### Case example

A 56-year-old male patient underwent a crush injury on the construction site, resulting in a tibial and fibular fracture in the right extremity (Gustilo-Andersen Type II, AO Type 42C2). Intramedullary fixation was implanted for tibial fracture as an initial internal fixation at the local hospital. After 6 months post initial surgery, right tibial nonunion was diagnosed. Infection signs of skin redness, swelling, increased temperature, and sinus effusion occurred in proximal one-third of the right tibia. He was then transferred to our trauma center for further surgical treatment. A swab bacterial culture result indicated MRSA infection. Systemic antibiotic treatment with vancomycin (0.5 g q12H ivgtt) was commenced with carefully monitoring renal function.

**Figure 1:**
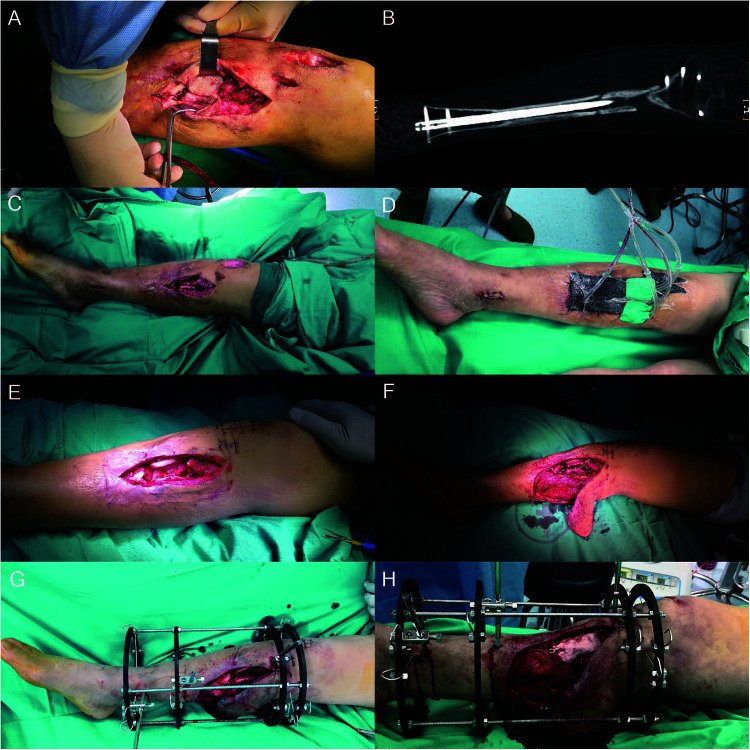
A 56-year-old male with a proximal one-third tibial infected nonunion, underwent the first stage procedure. The internal fixation was removed (**A,B**) and underwent radical debridement with VSD to cover the wound and cast for temporary fixation (**C,D**). After 5 days of drainage, removal of VSD showed fresh granulation tissue and no significant sign of infection (**E**). A randomized flap was used to cover the soft tissue defect (**F**), and ring external fixator (**G**) was used to stabilize bone ends. PMMA cement contained vancomycin filled up the dead space (**H**). VSD, vacuum sealing drainage; PMMA, polymethyl methacrylate.

For the first stage procedure, initial fixation was removed (Figure 1A,B). Nonviable tissues such as dead bones and infected sinus were all resected. VSD was used after complete debridement of the infected and necrosis foci (Figure 1C,D). After 5 days of drainage, removal of VSD showed fresh granulation tissue and no significant sign of infection (Figure 1E). Radical debridement resulted in a 4.0 cm bone defect with mono-cortical preservation. Vancomycin-loaded cement spacer was filled up with dead space and a randomized flap was applied to cover the soft tissue defect of sinus (Figure 1F). An external ring fixator was installed for bone stabilization (Figure 1G,H). The serological level of postoperative IL-6 has a significant decrease from 38.6 pg/ml to the normal range.

**Figure 2:**
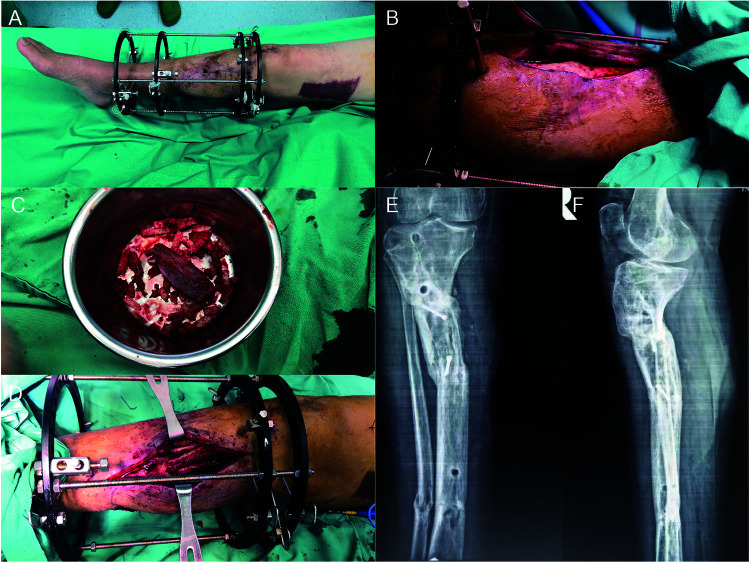
The second procedure of *de novo* osteosynthesis of nonunion bone. No sign of topical infection was observed before the second stage of surgery (**A**). Bone cement was removed with careful protection of induced membrane (**B**). The void was filled up with ABGs from iliac and allograft (**C,D**). Twelve months postoperatively, signs of osteointegration appeared and patients started weight bearing process. With dynamic motorization for 6 months, the external fixator was removed and no-protection weight bearing was achieved (**E,F**). The patient did not report any major complications.

For the second stage procedure, less than 5 neutrophils per high power field indicated the control of local infection, 5 weeks after the first stage (Figure 2A). Bone cement was removed and the void was filled up with iliac ABGs and allograft (Figure 2C). The iliac autologous bone graft was also used as a structural bone graft (Figure 2D). Twelve months postoperatively, signs of osteointegration appeared and patients started weight bearing process. With dynamic motorization for 6 months, the external fixator was removed and no-protection weight bearing was achieved (Figure 2E,F). The patient did not report any major complications.

## Discussion

The basic principle of bone nonunion treatment is widely accepted as the “diamond concept” to determine the mechanobiology considering soft tissue condition and bone stability (11). The diagnosis and treatment of infected bone nonunion are distinguished from infected segmental bone defect or osteomyelitis due to the focus on bone nonunion instead of critical size osseous reconstruction (1, 12). Until recently, infected bone nonunion remains a huge challenge than common nonunion, requiring radical debridement, susceptible antibiotic treatment, and suitable fixation (13). Lots of novel biological techniques have been performed in the preclinical studies of bone and joint infectious diseases (14, 15). Novel anti-infectious biomaterials mainly focused on the coating technique on scaffold biomaterials (16) or multiple antibiotics delivery system investigations (17). Nevertheless, these techniques still remained a long way from clinical translation.

At the beginning of the discussion, retention of implants has long been debated for a long time. For acute or early FRI of 3 weeks, implant retention might be considered for the stability of the bone fracture according to the type of implant and soft tissue conditions (4). When it comes to IBN, retention of the implant was not recommended. Recurrent infection was even reported in the Masquelet technique, which might be attributed to unthorough debridement and the possible bacteria retention in the implant (18). Thus, the removal of initial implant and the temporary fixation or external fixation during the first stage might be suggested instead of conventional fixation retention, especially in IBN. In our study, the initial implant was removed to create an unobstructed view of the bone and soft tissue for radical debridement.

From the perspective of radical debridement, the elimination of infected bone and soft tissue should be both taken into consideration in many studies (19). In terms of handling granulation tissue between the bone ends, a “paprika sign” (bleeding of the rest cortical bone) was required in each patient to make sure the removal of unviable infected bone (20). However, due to the treatment of infected bone nonunion, it is of great importance to debride the infection foci as completely as possible. In terms of soft tissue debridement, the surgeons should be full awareness of the balance between excessive and inadequate debridement. Benefiting from the Masquelet technique, the induced membrane provided a well vascular bed for surrounding tissue healing. Nonetheless, still one of the reinfected patients underwent reinfection due to the poor soft tissue condition after severe contamination of initial injury (Gustilo–Anderson type IIIC). In addition, lots of soft tissue reconstruction methods such as fasciocutaneous flaps have been combined with the Masquelet technique to achieve a local benign soft tissue coverage (21). Hence, the above-mentioned debridement details required experienced orthopedic surgeons to avoid recurrent infection and for later osteosynthesis procedures.

From the perspective of antibiotic treatment, the Masquelet technique has shown unique advantages in IBN. Our results have shown that the most common pathogen was MRSA in IBN, which was consistent in previous studies (9). The local recalcitrant infection resulted from low virulent bacteria forming key niches to escape from antibiotics through multiple mechanisms, including biofilm formation, canalicular invasion, and formation of staphylococcal abscess communities (22). The first stage application of PMMA of Masquelet technique filled up the dead space of the infectious cavities and could be used as a local antibiotic delivery system to eliminate infections. Although lack of evidence of recommendation for using local antibiotics, practical clinical use has shown satisfying results due to high local concentrations and fewer systemic side effects (23). Vancomycin was the most commonly used antibiotic used in PMMA to recover all-spectrum bacteria. The moderate dose (2 g) of vancomycin concentrate used in our study has little effect on osteogenic properties of induced membrane as previous study reported (24). Clindamycin has also been widely integrated into innovative biomaterials to show excellent antimicrobial properties in preclinical studies (25). The membrane-permeated antibiotic linezolid was also an effective strategy for intracellular *S. aureus* (26). Moreover, systemic administration of antibiotics was based on the results of the drug allergy test and the instruction from the international consensus (13).

From the perspective of suitable fixation, reconstruction of bone stability is versatile in IBN. For IBN treatment, due to worry of recalcitrant infection, external fixation of nonunion ends once was believed as the only method to treat infected bone union (27). Ilizarov technique not only stabilizes bone ends but also reconstructs the bone defect by bone transport procedure or limb length procedure (28). However, complicated instructions of bone transport, the discomfort of wearing fixators, and long period of bone consolidation largely affect patients’ daily living. Appeals to use internal fixation return back to surgeons' sight. With the resolution of incomplete debridement, early definitive fixation could even be performed when treating IBN with the Masquelet technique (29). Local benign soft tissue coverage and radical debridement enabled surgeons to use internal fixation for nonunion. Six patients in our study used internal fixation in the second stage, while 17 patients used external fixation in the first stage procedure. In addition, two patients even used external fixation of locking plate as patients not receiving external fixator. The autologous bone graft was adopted for structural bone graft, particularly in cases of mono-cortical bone preservation. No significant complaints were received from our patients. Nonetheless, the unique advantage of external fixation is the motorization procedure to accelerate the union of bone grafts. Although induced membrane of Masquelet has already enhanced osteogenic effect of local environment, the autologous bone graft is sometimes not sufficient for the bone union. The motorization procedure of the external fixation can help the grafted bone remodel through axial stress with recanalization of osteo-cavity and reestablishment of cortical continuity. Thus, the versatile use of fixation methods combined with Masquelet osteosynthesis might create a stable mechanobiological environment to achieve bone union.

Another new attempt in our study was the application of IL-6 as the infection indicator of IBN. IL-6 is a pleiotropic cytokine involved in immune responses, inflammation, and even bone metabolism (30). An increase in serum IL-6 levels is associated with trauma, infection, and surgery. After infection, IL-6 triggers the release of CRP and peaks earlier than that of CRP (31). In IBN patient, chronic infection stimulates the increase of infection indicator and decrease as long as the infection is removed. Our results have shown with the clear of infectious foci, IL-6 returned to the baseline faster than CRP and suggested no recurrent infection. The diagnostic value increased to 0.83 when compared with WBC, CRP, and ESR (7). WBC with a percentage and absolute value of neutrophil count showed low sensitivity and specificity for diagnosing IBN. CRP and ESR could be considered as infection indicators of infection (32). However, the problem with ESR, WBC, or procalcitonin is the fact that it can be normal in chronic infection patients, leading to poor accuracy in the diagnosis of joint and bone infection (33). IL-6 has a much less half-life, being four times shorter than CRP (34). The short half-life IL-6 serum marker is also consistent with the progressive trend of decreased length of hospital stay and rapid-recovery protocols (35). Thus, IL-6 has its own value in IBN diagnosis and prognosis judgment.

There remained several limitations in our study. The major limitation of the study is the small sample size of patients and having no setup of a control group. Another limitation is the versatile fixation method partially according to patients' prone, leading to heterogeneous results of different fixation methods. Nonetheless, our findings have provided sufficient evidence to show artifices of the Masquelet technique in treating infected bone nonunion. Future prospective comparative studies with other similar studies should be conducted to validate our experiences.

## Conclusion

The management of infected bone nonunion through the Masquelet technique with radical debridement and alternative fixation is reliable and feasible. The technique is effective for avoiding recurrent infection and osteosynthesis. A deeper understanding of the mechanism behind the technique should further be pursued.

## Data Availability

The original contributions presented in the study are included in the article/Supplementary Material, further inquiries can be directed to the corresponding authors.
